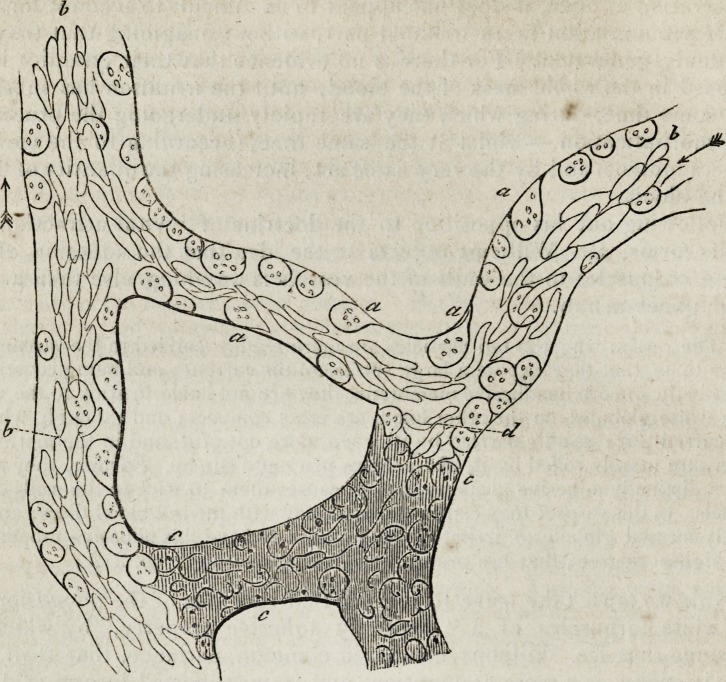# Principles of Medicine: Comprising General Pathology and Therapeutics, and a Brief General View of Etiology, Nosology, Semeiology, and Prognosis

**Published:** 1844-07

**Authors:** 


### ART. VI.

1. *Principles of Medicine : comprising General Pathology and Therapeutics, and a brief general view of Etiology, Nosology, Semeiology, and Prognosis.* By CHARLES J. B. WILLIAMS, M.D. F.R.S., Professor of the Principles and Practice of Medicine, and of Clinical Medicine; and First Physician to the Hospital, University College, London, &c.—*London,* 1843. 8vo, pp. 390. Article, *Inflammation.*
2. *The Actual Process of Nutrition in the Living Structure demonstrated by the Microscope ; and the Renewal of the Tissues and Secretions, with the Phenomena and Products of Inflammation, illustrated and established.* (*Second Series of Experimental Researches.*) By WILLIAM ADDISON, F.L.S, &c.—*London,* 1844. pp. 76. With two Plates.

DURING the interval which has elapsed since we last formally treated the subject of Inflammation, much addition has been made to our knowledge of the nature of this condition;—partly by means of the microscrope, which has furnished some new facts in regard to the condition of the corpuscles floating in the blood of the inflamed part, and in the products of the inflammatory action ; and partly by means of analytical processes of a simple kind, which have made certain that which was before only suspected, in regard to the altered proportions of the fluid elements of the blood. We think it time, therefore, to bring the subject again under the notice of our readers ; and the publication of the works named above affords us an advantageous opportunity for doing so. The general character of Dr. Williams's treatise, and most of the subjects embraced in it, have been noticed in our preceding Number ; but we have reserved the portion relating to Inflammation for more detailed discussion, —as well for the sake of combining with it a notice of Mr. Addison's researches, as because we think we can connect with it some views of our own, which may help to elucidate this most perplexing subject.

Our general appreciation of the merits of Dr. Williams's work has already been expressed ; and we need not, therefore, repeat it here. We shall only remark, that the portion now under consideration fully bears out the encomiums we passed upon the remainder; excepting that its value is impaired, in our estimation, by the adoption of the same notions, in regard to the forces which move the blood, as those we have already commented on in the sections on Congestion and Determination. The sketch of the whole subject, however, is drawn with the hand of a master; and on account, both of its scientific and practical value, is worthy of the most attentive perusal.

A tendency has manifested itself among many recent physiologists and pathologists (amongst whom Prof. Alison has taken the lead, as in so many other improvements), to regard the act of Inflammation as nothing else than an altered form of the ordinary Nutritive process. This, we are well convinced, is the true mode of viewing it. That it has not been so considered, and that attention has been so long and so exclusively fixed upon the state of the vessels, rather than upon that of the blood which they carry, and of the tissues which they penetrate, is, we are satisfied, the true explanation of the absence of profitable results, notwithstanding the vast amount of time and attention which has been given to the observation of the process. It is only by obtaining clear and positive ideas of the ordinary phenomena of Nutrition, that we can comprehend those alterations which, in a collective form, are spoken of as constituting Inflammation. Our knowledge of this department of physiology has received many accessions during the last few years; and the time seems to us to be arrived for its application to pathological inquiry. It will not be inappropriate, therefore, if we commence our exposition with a critical analysis of Mr. Addison's memoir; which we shall endeavour to treat with strict justice. We regret to find that our comments on his former paper were regarded by Mr. Addison and his friends as less favorable than its deserts required; but an attentive reconsideration of them, with all the aid derived from subsequent inquiry, and from the perusal of the present continuation, has only added increased force to our conviction, that the memoir to which we refer contained many statements and inferences, which will not stand the test of further investigation. And we cannot but think that our readers will be disposed to take a similar view, by the fact, that the present memoir is entirely devoted to the further elucidation of that subject, which we formerly noticed as the one in which we considered Mr. Addison's researches as most successful and satisfactory—the characters and functions of the colourless corpuscles of the blood. In bringing it under their notice, we shall offer no objection to the statements which Mr. Addison has recorded as *facts*—these being few, not inconsistent with generally-received doctrines, and easily verified. But we shall feel called on to inquire, how far the facts observed by him warrant the *inferences* which he has deduced from them. And if we do this with more strictness than the occasion may seem to warrant, we would say, in excuse, that the mode of reasoning adopted by Mr. Addison would, as we believe, if followed out in other directions, lead to the utter subversion of all philosophy.

The points which Mr. Addison considers himself to have established by his former researches on this subject, may be stated as follows:

1. That the colourless corpuscles exist in blood of man under all circumstances, and are constantly circulating through the capillary vessels, to the walls of which they have a tendency to adhere.

2. That they exist in great numbers in the blood of inflamed parts; and that they may be seen accumulating in the irritated vessels of a frog's foot, and showing an increased tendency to adhere to their walls.

3. That they exist in great numbers in the buffy coat of the blood.

4. That the liquor sanguinis—especially that of inflammatory blood—*fibrillates* in coagulating; so that a thin film of it presents all the structural characteristics and physical properties of fibrous or membranous tissue.

5. That lymph and pus-globules, exudation-cells, and epithelium, are altered forms of the colourless corpuscles.

With respect to the first four of these positions, we do not entertain the slightest doubt, since Mr. Addison's observations in regard to them coincide fully with those of other microscopists: and we fully agree with him in his estimate of their importance, believing that they will form the foundation of a novel physiological and pathological superstructure. But if we should venture to differ from Mr. Addison as to the plan on which this should be raised, we can only say that we desire our readers not to imagine us to affirm dogmatically that Mr. Addison is wrong, and that we are right; but merely to consider us as discharging our critical duty, in pointing out what we consider the weak points of Mr. Addison's argument. The *fifth* position taken by Mr. Addison, is the one to the "demonstration" of which this memoir is particularly devoted.

"By the application of several reagents," he tells us in his commencement, "and by comparing their effects upon the colourless blood-corpuscles, with those resulting from their application to pus-globules, I came to the conclusion that lymph and pus-globules, exudation-cells, and epithelium, originate from the colourless corpuscles. This conclusion was strengthened by the fact, that the fibrin of the liquor sanguinis was never seen, during the progress of fibrillation, to give origin to a corpuscle or globular particle of any kind.* The chief difficulty in establishing this conclusion arises from the doctrine, that all the blood-vessels have *permanent tubular parietes*. My object on the present occasion is to show the nature of these tubular parietes, the changes they undergo, and to point out how the colourless blood-corpuscles are included in their structure." (p. 3.)

The first point to which Mr. Addison directs our attention is the character of the normal fluid contents of the colourless corpuscles; and this he considers to be established by the following experiment, which we have ourselves repeated with a similar result:

"Provide six or eight slips of glass, such as are usually employed for mounting microscopical objects, and as many smaller pieces. Having drawn blood from a person with rheumatic fever, or any other inflammatory disease, place a drop of the colourless liquor sanguinis, before it fibrillates, on each of the slips of glass; cover one *immediately* with one of the smaller slips, and the others one after another, *at intervals of thirty or forty seconds;* then, on examining them by the microscope, the *first* will exhibit the colourless blood-corpuscles in various conditions, and numerous minute molecules distributed through a more or less copious fibrous network; and the *last* will be a tough, coherent, and very elastic membrane, which cannot be broken to pieces nor resolved into smaller fragments, however roughly or strongly the two pieces of glass be made to rub against each other. This is a 'glaring instance' of a compact, tough, elastic, colourless, and fibrous tissue, forming from the colourless elements of the blood: and the several stages of its formation may be actually seen and determined. Numerous corpuscles may be observed in all these preparations to have resolved themselves, or to have fallen down into a number of minute molecules, which are spread out over a somewhat larger area than that occupied by the entire corpuscles; and although still retaining a more or less perfectly circular outline, yet refracting the light at their edges in a manner very different from that in which the corpuscles themselves are seen to do. It is from these and various other larger and more irregular masses of molecules or disintegrated corpuscles, that the fibrinous filaments shoot out on all sides, as from so many centres; or frequently the filaments are more copious in two opposite directions. It is highly probable that the molecules and the plastic fibrillating liquid are both derived from the interior of the colourless blood-corpuscles." (pp. 4-5.)

From this and other corresponding observations, Mr. Addison draws the conclusion, that neither the fibrin or albumen of the circulating blood

* In reference to this statement, we would remark, that Mr. Addison's observations seem to have been made upon fibrin withdrawn from the body, and coagulating upon a *dead* surface. Under such circumstances, it is not surprising that the cell-germs which the fluid may contain, should not be able to develope themselves into cells; but the history of the organization of fibrinous effusions poured out upon *living* surfaces seems to show that such a development *may* and *does* take place.

are diffused through its fluid portion, or liquor sanguinis; but that they are both contained in the colourless corpuscles; of these he imagines that a large proportion burst or become ruptured immediately that the blood is drawn from a vein, owing to the sudden change of temperature to which they are exposed, or from other causes; and that they set free the liquor sanguinis, which rises to the surface, drawing up with it the colourless corpuscles which have hitherto preserved their integrity:

"Accordingly, neither the fibrinous element nor the serum circulates in the blood, as part of the fluid in which the red corpuscles are suspended in the living vessels; they are both inclosed within, and form, incorporated together, an essential ingredient of the interior contents of the colourless corpuscle. Hence, therefore, the fibrin can no more be said to be elaborated from the albumen, than the stone or kernel of a peach can be said to be elaborated from the fruity pulp—they both grow together; so likewise the fibrinous element and the albuminous element grow or are elaborated from other sources: if they bear a due proportion to each other, the corpuscle or cell will be normal, and its subsequent function will be healthy; but if one element preponderate over the other, the reverse of this must happen. What, then, it may be asked, is the nature of the liquid in which the corpuscles move when in the living vessels? It is impossible to determine: for it cannot be procured for experimental examination without being mingled with corpuscles, nor, according to these views, without some of them bursting and mingling with it their own contents." (pp. 7-8.)

The startling doctrine here enunciated is somewhat qualified in the following note, attached to the end of the memoir: "It is not intended to deny that the fluid in which the blood-cells float, in their passage through the living vessels, is not, or may not be, an albuminous fluid; all that is meant by the affirmation is, that it is *not serum*, though clearly it must be mingled with, and form part of the bulk of this fluid." By this we presume that we are to understand, that *a part*, if not the whole, of the albuminous element of the serum is derived from the rupture of the colourless corpuscles; and that *all* the fibrin of the circulating blood is contained in them. Now, upon this we shall simply remark, that the doctrine seems to us entirely hypothetical; to be *not required* by any of the *facts* adduced by Mr. Addison; and to be inconsistent with many others, very well established. For the experiment we have quoted, and others of a similar character, only go to render it highly probable (for we scarcely think that they demonstrate) that the fluid contents of the colourless corpuscles, which they yield in bursting, are of a peculiarly *plastic* or organizable character; in other words, that they are of a *fibrinous*, rather than of an *albuminous* nature. They do *not* prove that *all* the plastic matter of the liquor sanguinis is contained by them, whilst the blood is circulating through the living vessels; still less that any considerable proportion of albumen can be shut up within them. And that such is the case, seems to us altogether disproved by a comparison of the relative amounts of the different elements of the blood. For, on watching the circulation in the living animal, the number of colourless corpuscles which traverse the field is by no means sufficient to hold the quantity of fibrin and albumen (probably not even that of the former alone), which we know to exist in the amount of blood that has flowed through the vessels during our observation. For it must be remembered, that the contents of these corpuscles are *fluid;* and that the numbers which are used to represent the proportion of fibrin in the blood, refer to its *dry* state, in which its bulk is of course far less. Mr. Addison further objects to the doctrine, that fibrin is elaborated from albumen; and speaks of them as both elaborated together, from other sources. We were not aware that albumen needed *any* elaboration; having always been accustomed to regard it as derived immediately from the food; and until Mr. Addison can show us that the albumen of the blood differs from that of the chyle, or that the fibrin is derived from any other source than the albuminous element, we shall take leave to consider it as an established position, that the fibrinous element of the blood is nothing else than albumen in process of preparation or being organized; that is, becoming endowed with plasticity and certain other peculiar properties which albumen does not possess. The doctrine, that the elaboration of the plastic element is performed by the agency of the colourless corpuscles, which was first propounded by Dr. Carpenter in this Journal, (vol. XV, p. 273,) seems to us very probable; and our readers can scarcely fail to perceive what striking confirmation it receives from the experiment we have just quoted. We believe that most physiologists, who are not prejudiced by the seductive simplicity of Schwann's generalization as to the derivation of all tissues from cells, are now arrived at the conclusion that, as regards the areolar and other simple fibrous tissues, no other explanation of their production need be looked for, than the known tendency of the particles of fibrin to arrange themselves in a linear manner, so as to form fibres; which tendency manifests itself much more decidedly, when the consolidation takes place upon a living surface, than upon a dead one. Mr. Addison's observation on the mode in which this takes place in buffy blood, seems to us to give a most satisfactory explanation of those appearances, which have led Henle and other microscopists, who did not coincide with Schwann in the idea that the fibres are derived from *cells*, to assert that they were produced from the *nuclei of undeveloped cells.* In the assistance which he has given towards making these important corrections, we regard Mr. Addison as having done good service to physiology.

Mr. Addison next proceeds to explain his views of the relation between the *liquor sanguinis* (or, according to him, the normal contents of the corpuscles), and *mucus* and *pus.* He states, that not only may we see in mucus numerous molecules and colourless globules, but that it frequently exhibits a copious and distinctly fibrous character or structure, and always does so when a little dilute *acid* is added to it—an addition which cannot *form* the fibres. On the other hand, if a few drops of the liquor sanguinis from buffy blood, before fibrillation, be treated with *alkali* (liquor potassæ), it becomes quite clear, transparent, and colourless, in consequence of the rupture of all the white corpuscles; and the mixed materials become semi-solid, and resemble a glairy mucus. Further, if a drop of blood be treated in a similar manner, the red corpuscles also rupture, and the resulting liquid has a transparent, brownish tint. On subsequently mixing a drop of dilute acetic acid with either of these mucus-like liquids, it is rendered opaque, and displays a more or less evident fibrous arrangement, according to circumstances. When a drop of white and opaque healthy pus is placed upon a slip of glass, and is well mingled with a drop of liquor potassæ, it entirely loses its opaque character, and becomes clear and transparent, resembling mucus; with such a degree of tenacity and elasticity, that it may be drawn out into strings or filaments, six or eight inches long. On placing it under the microscope, it is seen to contain numerous molecules and granules, but no entire corpuscles, all having been ruptured by the alkali. But on mingling a drop of dilute acetic acid with this, it shrinks up, and resumes somewhat of its former opaque, white aspect; and on submitting it to an examination by the microscope, when pressed into a thin film, it exhibits as copious a fibrous structure or character as the fibrillated liquor sanguinis. Similar observations are added at the close of the memoir, in regard to the changes produced in animalcules, and in certain cells from the earthworm, by the agency of liquor potassæ and acetic acid. Mr. Addison adds some other observations, in regard to the effects of reagents upon the three kinds of corpuscles respectively; and from the similarity of these actions, he draws the conclusion, that pus- and mucus-globules are nothing else than altered forms of the colourless corpuscles of the blood. The following quotation fully enunciates his conclusions on this subject:

"1st. That the plastic fibrillating liquid, denominated liquor sanguinis, exists as a fluid within the colourless blood-corpuscles, and that when it escapes from them it forms an elastic fibrous tissue, the serum being the residual liquid.

"2d. That mucus- and pus-globules are altered colourless blood-corpuscles; and that the glairy fluid termed mucus is nothing more than an altered state of the fibrillating liquor sanguinis; the change from one to the other being coeval with the changes which characterize the microscopical aspect of the corpuscles. Hence, if we take the red portion of the buffy clot, and the red blood-corpuscle, to represent blood, then the colourless layer of liquor sanguinis, with the colourless blood-corpuscle will represent the first remove from blood, and mucus or pus, with the mucus- or pus-globule, will be the next. And it would appear generally, that the nearer the corpuscle is to, or the fewer the stages of its removal from, the circulating fluid, the more nearly it resembles the colourless blood-corpuscle, and the more decidedly and visibly its fluid contents, when they escape, fibrillate; whereas, the further the corpuscle is, or the greater the number of stages of its removal from the circulation, the larger it is, the more it is filled with molecules, and the less perfectly do the fluid contents fibrillate. Now, if it be admitted—of which I have myself no doubt—that the fibrillating liquor sanguinis is changed into mucus in the interior of living cells, then there can be no difficulty in admitting that similar living cells, by a different mode of elaboration, may not only form sundry kinds of fibrous or mucous tissue, but the tears, saliva, milk, or bile." (pp. 15-16.)

By these and other observations, Mr. Addison has been led to entertain the following new doctrine of Secretion:—that the cells by which (as it is now generally admitted) the secreted products are elaborated, are not developed in the secreting organs themselves, at the expense of materials supplied by the blood; but that they are neither more nor less than the colourless corpuscles of the blood, which elaborate these products while still floating in its current, and escape from the vessels in a manner to be presently explained. He appears to think that in this mode he gives a better account of the matter, than that afforded by the ordinary doctrine; which attributes the separation of particular products by particular glands, to "some peculiar transcendental and purely hypothetical selective process of exudation, through a structureless and transparent tissue." As we are of an entirely different opinion, we shall endeavour to make clear to our readers the grounds on which we think that the doctrine has no claims to acceptance.

It will be useful, as a preliminary, to arrange the products of secretion under two heads: ***first,*** those which result from the decompositions constantly taking place within the body, and which must be eliminated from the blood, in order to preserve its purity; and *second*, those which are intended to serve some particular purpose in the economy. The chemical characters of the former group—including the bile, urine, and the fetid secretions of the intestinal glandulæ—are entirely different from those of the blood; and it cannot be imagined that they can result from any simple metamorphosis of its elements. Moreover, if their elimination be checked, they accumulate in the blood, and then find their way out of the vessels, through other tissues and surfaces, by a simple physical process of exudation. But the fluids of the latter class, such as the lacrymal, salivary, pancreatic, and mucous secretions, do not differ from the blood in anything like the same degree; and may be easily conceived to be generated from it by a simple action, such as that by which Mr. Addison produces a kind of artificial mucus from the liquor sanguinis. They do not seem to accumulate in the blood, if their production be checked; and, consequently, there does not seem any occasion for supposing them to be pre-formed in it. All are agreed that the elimination of both kinds of secretions from the blood is a consequence of cell-growth: but the general opinion is, that cells developed in the substance of the liver have the power of selecting biliary matter; that cells developed in the tubuli uriniferi have the power of selecting urinary matter;—and so on of other glands. Now, on Mr. Addison's hypothesis, (for such we must term it, since there is not a shadow of *direct* proof offered by him—the only argument in its favour being the resemblance between certain characters of some of these secreting cells, and those of the colourless corpuscles of the blood,) we must regard the blood as containing not only the elements of all these secretions ready formed, but also the cells which eliminate them; and that, of the corpuscles which are commonly ranked under the appellation, "colourless," some are engaged in elaborating bile, others urinary matter, others milk, &c. Here, then, is a process of selection just as "transcendental" as that which is commonly supposed to take place in the glands themselves, and a great deal more hypothetical.—But further, it is necessary to suppose, on Mr. Addison's theory, that all the cells concerned in the separation of biliary matter find their way out through the liver, in virtue of some peculiar power which determines them to that organ;—that all the cells which secrete urine find their way out through the kidneys, for a similar reason; and so on of other glands. Hence, then, Mr. Addison's theory really requires *two* sets of "selective processes:" one by which the cells take up the product to be separated, and the other by which the gland takes up its particular class of cells;—whilst other physiologists are satisfied with but one. But he may affirm that we have misrepresented his opinions, in the assumptions to which we have shown that they lead; for that he would not assert that any of the colourless corpuscles floating in the blood hold the products of secretion, but rather that they become charged with them in passing out of the current through the glands. This *might* be admitted (if other circumstances rendered the hypothesis favorable) in regard to the secretions of the second class; as it might be supposed that the liquor sanguinis and colourless corpuscles became changed, in passing through a mucous membrane, into mucus and mucus-corpuscles, by some special influence there exerted; or into salivary matter, when passing through the salivary glands. But it *cannot* be admitted in regard to the bile and urine—of the pre-existence of whose elements in the blood, we have the most abundant evidence. Either, therefore, these matters must be contained in some of the corpuscles of the circulating blood, which is a necessary part of Mr. Addison's doctrine of secretion; or they must form part of its fluid ingredients, and must be separated from the liquor sanguinis by the cells developed within the several organs through which they are eliminated. In support of the latter doctrine, it appears to us that every analogy may be urged; besides the palpable objection which must occur to every one in regard to the former,—that we have no ground whatever to suppose that the secreting cells pass out *bodily* from the capillary vessels, and find their way through the basement membrane of the tubuli or follicles, impervious, as it would appear, to take their place as epithelium-cells on their lining.

We cannot find that any argument in favour of this doctrine is supplied by Mr. Addison's researches, save that which rests upon the similarity of the colourless corpuscles and the epithelium-cells, as to their aspect when viewed with the microscope, and their behaviour (as the chemist would say) when treated with certain reagents, especially the liquor potassæ. Mr. Addison objects to an observation in our critique on his former Researches, to the effect that there is something too specific in the *character*, if not in the *appearance*, of pus-globules, to admit of the conclusion that they are altered colourless corpuscles; and he remarks, "the only possible way that I am aware of, in which pus-globules can present a sensible character, is through the medium of the microscope," which renders it "impossible to separate, as regards these minute objects, *character* from an *appearance*." (p. xix.) Now, the distinction between the two seems to us obvious enough; most objects having a number of *characters*, of which their *appearance* to the eye presents to us but a small proportion. And we consequently maintain, that we have no right whatever to assert the *identity* of such objects as those we are now concerned with, by their *appearance* only; and that we must take into account their other *characters*. We think it would be quite possible to place before Mr. Addison a dozen cells, among which he should not be able to distinguish the slightest difference, either as to microscopic *appearance*, or as to behaviour with liquor potassæ or other reagents. And yet the *characters* of those cells might be most completely and specifically different; for one might be the germ of a zoophyte, another that of an oyster, another that of an insect, another that of a fish, and so on, up to man; each cell requiring a certain peculiar set of conditions for its development, and producing a certain definite structure when supplied with those conditions. On Mr. Addison's mode of reasoning, all these cells must be identical in *character*, because his microscope does not reveal to him any distinguishing appearances amongst them; the cell which forms the zoophyte, therefore, should be able to produce a man, if placed in the appropriate circumstances; and the germ of man might be degraded to the production of a zoophyte. We hope that this "glaring instance" may satisfy Mr. Addison that our distinction is *not* without a difference; and that he may now be prepared to understand our objection to considering the various kinds of epithelium-, pus-, and mucus-corpuscles as altered forms of the colourless corpuscles of the blood. It seems to us, that if we find one set of cells invariably elaborating a certain product, and another set constantly elaborating another product, a distinctive *character* is thus furnished to us between the two; which should outweigh all that the microscope can reveal to us in regard to their identity of appearance. For, to assert that the microscope is able to reveal to us the whole history of cell-life, and to enable us to determine, by simple inspection, what is the function which any given cell is destined to perform, is to claim for it, as it seems to us, a most undue degree of power as an instrument of research. We have lately been comparing, with some degree of attention, the various forms of epithelium- and cancer-cells; and we have come to the conclusion, that while some of these forms are sufficiently unlike one another to enable them to be separated by a definition, others are so nearly allied as to render it impossible to determine, from their appearances, to which group they belong. Yet every one knows that there are *characters* by which the two are distinguished—cancer-cells being remarkable for their extraordinary reproductive power, which gives the character of malignancy to the tissue they generate; whilst epithelium-cells have no such capability. Yet, upon Mr. Addison's system of reasoning, a tumour consisting entirely of accumulated epithelium-cells (and such we have seen) must be regarded as cancerous in its nature.

We trust that we have now sufficiently explained ourselves upon this point, and that we have shown the fundamental error of Mr. Addison's reasoning. As we have before remarked, we set a high value upon his *observations;* and we hope that it is something better than the influence of pre-formed opinions, which leads us to object to his inferences. We must now inquire into the evidence on which Mr. Addison rests his assertion, that the colourless corpuscles escape from the vessels, for the purpose either of nutrition or secretion. As far as we can gather from his observations, it is the following.—In the capillary vessels of the embryo, there is no definite lining membrane; but altered colourless corpuscles are seen incorporated with the fibres which form their walls. This statement is illustrated by a very beautiful drawing of a blood-vessel from a fœtal hare. In the transparent larva of an insect, a similar appearance was noticed by Dr. Carpenter, and communicated by him to Mr. Addison. In regard to these two facts, we must object to their being used to prove anything in regard to adult structure; since it is well known that, at the commencement of the formation of the vessels in embryonic structures, (those of the larva of an insect ranking under that category,) they are mere intercellular passages, without any definite walls whatever; and that these are gradually formed, during the same time that a process of metamorphosis is taking place in other structures. The next class of facts adduced by Mr. Addison, has reference to certain appearances seen in the adult structure. "Colourless corpuscles," he says, "and little masses of detached molecules, are found incorporated among fibres in all growing membranes, in the fibrous walls of the blood-vessels, in the buffy coat of the blood, and in mucus. There is a gradual transition between the colourless blood-corpuscles in the interior of the blood-vessels, and the lymph- and pus-globules, the exudation and epithelial cells on their exterior." (p. 28.) That the cells within the vessels, and the cells on their exterior, are identical, is, as we have already endeavoured to show, just the thing *to be* proved. The assumption that they *are* so, appears to us but a very poor foundation for the inference, that the walls of the capillary vessels have pores large enough to allow these bodies to pass out. The utmost extent to which Mr. Addison has witnessed any such process, appears to be that specified in the following quotation :

"But it may be urged, as an objection to this theory of nutrition, that as we *can see* the colourless corpuscles in the irritated web of a frog's foot adhering to the tissue, why *do we not see* them passing through it, and forming fibres or epithelium ? To this I answer, that the nutritive changes or processes are too slow in this example for us to follow, from beginning to end, all the actual stages of nutrition ; the corpuscles go on congregating in the irritated tissue for an hour or two. Nevertheless, the epithelium of the web and the walls of the capillaries have their visible characters gradually changed during the observation ; and numerous corpuscles, more or less altered in shape, may be seen mingled with or buried in the living tissue. This is as far, perhaps, as we are able to go in *seeing* the process of nutrition ; and yet, if the part were patiently watched for a sufficient length of time, it is very possible that still more of the process might be seen. Moreover, that the great accumulation of the lymph-globules or colourless blood-corpuscles in the capillary channels of the web of the frog's foot, after immersion in warm water, their adhesion to, and incorporation with the tissue, is a 'glaring instance' of an acceleration of the normal process of nutrition, is in my opinion, substantiated by the fact, that, in the course of a day or two after the application of the warm water, the cuticle or epithelium of the web, composed of pentagonal or hexagonal scales, with their well-known nucleus in the centre, looking like a lymph-globule, peels off in large flakes—an event perfectly according with the theory, as it would not have happened so speedily, except for the application of the irritant." (p. 32.)

In like manner, the exfoliation of the skin in scarlatina is regarded by Mr. Addison as due to an accumulation of the colourless corpuscles in the blood, in consequence of the disorder of the processes of nutrition and secretion ; and he thinks that these cells are thrown off from the surface, either by a desquamation of cuticle, or (in the less favorable forms of the disease) by muco-purulent discharges from the internal epithelial surfaces. A number of other well-known facts are adduced by him, in support of his doctrines ; but we feel justified in affirming, after an attentive examination of them, that they are at least equally consistent with the doctrines commonly received, as to the presence of morbid matters in the blood. And we shall leave it to our readers to determine whether Mr. Addison can be said to have "demonstrated" the process of nutrition in the living structure, as he conceives himself to have done.

We shall allude to but one more topic before we dismiss Mr. Addison's memoir ; namely, some observations on which he lays great stress, and which are certainly of much interest, relatively to the *motion* of the molecules contained in the colourless blood-corpuscles, in pus-cells, and in epithelium-cells. These motions have been frequently seen by him, when the cells have been made to discharge their contents by means of liquor potassæ ; and he has also noticed them, in many instances, in the molecules still contained in the interior of the cell. Thus, in the mucous globules found in saliva, he has seen myriads of molecules in the most active state of motion, "almost reminding one of the busy scene of an ant-hill:"

"The globules in which this remarkable appearance presents itself are always perfectly circular, and have a uniform molecular aspect, free from any conspicuous granules, nuclei, or discs. This observation has directed my attention more particularly to the cause of the innumerable and various appearances presented by pus-globules; and I now believe that the irregularity in shape and outline, and the appearance of conspicuous granules, nuclei, or discs, either in these globules, or in mucous globules, is connected with the cessation of the active movements of these minute molecules. The perfectly-circular and uniformly-molecular mucous globule is a living cell; while those globules presenting the characters which have hitherto been considered characteristic of pus-globules, are, in my opinion, dead ones." (p. xvii.)

Such motions are by no means uncommon, especially in the cells of vegetables. We have ourselves repeatedly witnessed them; and fully agree with Mr. Addison in regarding them as altogether different from those molecular motions, which most minute inanimate particles exhibit under the microscope. We are inclined to think that they might be found in *all* cells, at a certain period of their development; and we cannot therefore regard their existence as of any value in identifying cells, whose other characters are different, with each other.

We shall now express, in a concise form, our own views of the nature of the Nutritive Process, in such a form as to admit of comparison with the abnormal condition of that process in Inflammation.

I. *a.* The blood supplies to the solid tissues the materials of the reconstruction, which is continually going on during their life; as well as the elements of their secretions, whether natural or morbid. *b.* The *fibrin* of the blood is the chief, if not the sole material for the formation or reconstruction of tissue; being, in fact, nothing else than *albumen in the act of becoming organized.* The change by which it is produced is one of a peculiarly *vital* nature. *c.* Fibrin, when duly elaborated, has the power of spontaneously assuming, by its coagulation, (which must be regarded as an act of vitality,) an organized form, of a low character—simple fibrous tissue. This *organizability* or *plasticity*, therefore, is the distinguishing property of fibrin. *d.* On the presence of this element in the blood, depends its capability of supplying the materials for the formation of new tissue; and the plasticity of the blood, taken as a whole, depends upon the proportion of well-elaborated fibrin which it contains. *e.* The elaboration of fibrin is an act performed by the *white* corpuscles of the blood, which bear a pretty constant relation to it, in their proportional amount, under different circumstances. *f.* The *red* corpuscles are to be regarded as chiefly, if not solely, ministering to the function of respiration; and as not essentially connected with the act of nutrition, otherwise than by supplying oxygen to the tissues and to the other constituents of the blood, and by conveying away their carbonic acid. *g.* The *albuminous* matter of the blood may supply the materials for certain secretions; but it does not immediately assist in the production of tissue, being in itself unorganizable or aplastic. *h.* The

*liquor sanguinis* of the circulating blood, containing the fibrin as well as the albumen in solution, is the portion of the fluid essentially connected with the formative processes. Its fibrin is being continually withdrawn from it, in the healthy state of the body, by these actions; and is being resupplied as continually, by the elaboration of the albuminous material, through the agency of the white corpuscles.

II. *a.* The various solid TISSUES which are in continual process of change, more or less rapid, derive the materials of their reconstruction from the blood, especially from its fibrin; which they have the power, by their vital endowments, of causing to assume their own respective forms of organization. *b.* The vitality of the tissues in any part may vary in its degree; so that their formative power may be increased or diminished. When their formative power is increased, the process of nutrition is performed with unusual rapidity, and the fibrin of the blood is rapidly drawn from it; but when the formative power is diminished, the process of reconstruction is slowly and imperfectly performed, and the demand for fibrin is less. *c.* When their vitality is still further depressed, they will have an unusual tendency to disintegration, and but little disposition to re-formation; and when it is altogether lost, the process of renewal is completely checked, and destructive decomposition takes its place. *d.* Where *new* parts are being formed—as in the reparation of injuries—a portion of the fresh tissue is the product of the effused fibrin, which becomes fibrous in coagulating; but another portion is the result of cell-growth; and the germs of the cells, as well as the pabulum by which they are developed, are probably effused from the blood.

III. *a.* The MOTION OF BLOOD in the capillary vessels is dependent, not merely upon the propulsive power of the heart and arteries, but also upon forces generated in the capillaries themselves, by the actions to which the blood is subservient whilst passing through them. These forces appear to be of such a character, that the several particles of blood are *attracted* towards the points where they are destined to undergo a change, and *repelled* from them after they have been subjected to it. *b.* If these actions, whether of nutrition or secretion, be unusually energetic, the motion of the blood will be accelerated; and much more than the ordinary amount of blood will pass through the part in a given time, constituting *determination* of blood, or *active congestion.* *c.* But if these actions be retarded, there will be a diminished rate of motion in the blood in the capillaries, constituting *passive congestion;* and if they be altogether checked, there will be an entire *stagnation* of its flow through the part, notwithstanding the impulsive action of the heart. *d.* These actions may be interrupted by a want of their proper conditions, on the part either of the blood or of the tissues, or of both.

IV. *a.* The PRODUCTS of the *normal* actions that take place between the blood and the tissues through which it moves, are either *new tissues*, of a character similar to that of the respective parts through which the fluid moves; or the *secretions* peculiar to those parts respectively, which are themselves elaborated by cell-growth, and therefore by an operation that may be considered as part of the general formative processes. *b.* When these processes are unusually active, in the state of *determination* of blood to a part, the products are the same in kind, but larger in amount. *c.* When these processes are slowly and feebly performed, as in *congestion*, the products are still the same in kind, but are less in amount. The obstruction to the circulation of the blood, however, causes a mechanical transudation of its more fluid portions, through the walls of the vessels, into the surrounding tissues, or upon the neighbouring surfaces; in this manner effusions of *serum* may take place to a considerable extent; and it has been lately shown that, when the obstruction is complete, or nearly so, even *fibrin* may thus find its way out of the blood-vessels. *d.* In the formation of new parts, as in the reparation of injuries, an increased production of fibrin in the blood may take place by local action; but if this fibrin be drawn off as fast as it is formed, there will be no accumulation of it in the blood; and, consequently, if no morbid products are formed, such processes *may* be performed without inflammation.

We shall now put into the same form the positions which we think may be assumed in regard to INFLAMMATION; with the intention, however, of presently dwelling upon many of them at greater length. We wish to be understood as here referring to the *sthenic* form of this process: the peculiar characters of *asthenic* inflammation will be hereafter noticed.

I. *a.* The quantity of *fibrin* in the BLOOD undergoes a decided increase; the *plasticity* of the whole mass, therefore,—but especially that of the liquor sanguinis,—is greatly augmented. *b.* There is a corresponding increase in the proportion of *white* corpuscles; which are present in large amount in the vessels of the inflamed tissues, and have a great disposition to adhere to their walls; but which are also present, to an unusual amount, in the entire mass of the circulating blood. *c.* The increase in the proportion of fibrin is chiefly a local action, exerted on the blood during its passage through an inflamed part; and probably effected by the instrumentality of the *white* corpuscles. *d.* There is usually an increase, not only in the *quantity* of fibrin, but in its *plasticity* or tendency to become organized; as shown by the greater perfection of the fibrous structure into which it passes in coagulating. This may consist in an increased attraction between its particles; which continues to operate for some time, causing contraction of the fibrous network, subsequently to its first production. *e.* There is also an increased attraction between the red particles of the blood; causing them to adhere together in rolls, more firmly and for a longer period than they do in healthy blood. *f.* To these two causes, usually aided in their operation by the slowness of the coagulation—all concurring to produce an increased tendency to separation between the red corpuscles and the liquor sanguinis,—we may ascribe the production of the buffy coat of inflammatory blood. *g.* The increased *plasticity* of the blood is so constant a phenomenon of inflammation, that it may be regarded as essential to the presence of that state.

II. *a.* On the other hand, the *formative power* of the inflamed TISSUES appears to be diminished; their usual functions, whether of nutrition or secretion, being completely checked, or insufficiently performed, or perverted in their character. *b.* Whilst, therefore, an over-production of fibrin is taking place in the blood, there is diminished consumption or appropriation of it in the tissues. *c.* If the inflammation be severe in its character, the vitality of the tissues is so diminished, as to cause not only a cessation of their formative actions, but also an increased tendency to disintegration, as shown in *suppuration* and *ulceration;* or positive death of a large part, as in *gangrene. d.* The depression of the vitality of the tissues sometimes appears to result from a previous over-excitement of it; as when inflammation follows excessive use of a part, or the application of stimulants to it: but it is sometimes the consequence of some directly sedative action, as that of cold. *e.* Hence both *determination* of blood, and *congestion*, have a tendency to produce inflammation: the one being a state of over-excitement, which is very prone to occasion subsequent depression, whilst there is at the same time a tendency to increased production of fibrin in the blood; the other being itself a state of depression of formative power in the solids, but not passing into inflammation, unless there be at the same time an increased plasticity of the blood.

III. *a.* The MOTION OF THE BLOOD in the capillaries of the inflamed part is greatly retarded; as we might have anticipated from the impairment of the functional operations of the solids. There may even be a total stagnation of the blood in the capillaries of a considerable portion of the tissue; which will be followed by its death and disintegration. The degree of stagnation will depend upon the amount of the depression of the vitality of the surrounding parts. *b.* The motion of blood through the vessels in the neighbourhood, however, is more rapid than usual, and these vessels are themselves enlarged; so that the total quantity which passes through an inflamed member in a given time is greater than usual. *c.* The vessels are enlarged both *in* and *around* the inflamed part, in consequence of a diminution of the tonic contractility of their walls; which causes them to admit of abnormal distension, by the impulse which the blood receives from the heart. This diminution is another evidence of the depression of the vital properties of the solid tissues in an inflamed part.

IV. *a.* The PRODUCTS of inflammation differ from those of the ordinary processes of nutrition and secretion, not so much in their materials, as in the nature of the change which these have undergone. *b.* When the intensity of the inflammatory process is moderate, the *liquor sanguinis,* containing an unusual proportion of fibrin, and possessing a high degree of plasticity, is effused into the neighbouring tissues, or upon the neighbouring surfaces; being generated, by the local actions of the part, faster than it can be withdrawn by its formative processes. By the organization of which it is susceptible, when in contact with the living solids, it spontaneously assumes the form of simple fibrous tissue, constituting false membranes on the surface, or consolidating the substance into which it is effused. *c.* If the inflammatory process goes no further, there is no disintegration of the original tissue ; but if its vitality be too far depressed, it dies ; and the changes which it consequently undergoes, impress themselves upon the fibrinous effusion. The fibrin loses its vital power of coagulation, and in this aplastic state becomes the chief ingredient in the *liquor puris ;* whilst the cells (*pus-corpuscles*), which are found floating in it, resemble the white corpuscles of the blood in a degenerated form. *d.* When the inflammation is very severe, and the stagnation of blood in the capillaries of the part is complete, an entire loss of vitality in the whole tissue at once, or *gangrene*, is the result. Gangrene does not originate, however, in inflammation alone; since any other cause, such as the long-continued action of cold, or pressure, interrupting the capillary circulation, or obstruction to the supply of blood through the arterial trunks, will equally produce it, by the suspension of the formative processes thus occasioned. But unless some degree of inflammatory action, that is, an increase in the plasticity of the blood, be set up at the same time, there is an indisposition to the formation of the line of demarcation between the sound and the dying parts, and the gangrene has a tendency to spread.

We shall now enlarge upon some of these doctrines ; and advert to the evidence upon which they are founded.

The large increase of fibrin in the blood of persons labouring under acute inflammation, has been sufficiently proved by the experiments of Andral and Gavarret ; a similar statement having been made, however, by previous observers; though upon insufficient grounds. The phenomenon is so constant, that it must be regarded as a pathognomonic sign of inflammation, distinguishing it from other conditions which simulate it ; and enabling it in some instances to be discovered at an earlier stage, than that at which it could have been detected by either general or local signs. The degree of the increase bears a constant proportion to the extent of the inflamed part, and to the intensity of the morbid action. The proportion of fibrin has been observed in some cases to amount to as much as 10 parts in 1000, the normal proportion being from $2\frac{1}{2}$ to $3\frac{1}{2}$ ; and even when it was previously lower than usual, as in continued fever, it began to rise as soon as ever a local inflammation developed itself. That the increase is not only in the quantity of fibrin, but in its plasticity (or tendency to become organized) also, appears from various phenomena of inflammation ; such as the rapid production of false membranes from fibrinous effusions; as well as from the more complete fibrous arrangement seen in the buffy coat, than that which the ordinary coagulum of blood displays.

The increased proportion of the white or colourless corpuscles, in inflammatory blood, and their special accumulation in the vessels of the inflamed part, is the concurrent result of the independent observations of Gendrin, Gulliver, Addison, and Williams. The observations of the first two of these gentlemen are chiefly directed to the increase of these bodies in the *whole mass* of the blood ; the two latter have also directed their attention to the part they play in the production of local changes; and although their observations were made in each case without the knowledge of the results obtained by the other, the harmony between them is most complete, and therefore gives us a strong impression of their accuracy, more especially as many of them have been confirmed by our own observations. As this is a point of great interest, we shall extract Dr. Williams's statement in full. We should premise that he accords with other observers, (Mr. Wharton Jones and Mr. Addison for example,) in the remark, that, in the ordinary state of the circulation of the frog, the white corpuscles have a tendency to adhere to the sides of the blood-vessels, remaining in the almost motionless layer of liquor sanguinis that surrounds the rapid current in which the red particles move.

"I have never seen a solitary elliptical disc adhering to the sides of a vessel; and whenever one was arrested in its course, it was from its becoming hitched by one or more of the adherent round globules. But what appeared to me most remarkable in regard to these white globules, was the great difference in their number under different circumstances. In young frogs, and in those much subjected to experiment, they are always present: but in healthy adult frogs, placed under the microscope with as little handling of the web as possible, there were few or none to be seen. I have watched, for ten minutes at a time, without seeing one; the motionless layer was very thin, but clear, and all the blood particles in the larger vessels seemed to move at the same rate of speed. It is under these circumstances that the effect of irritation or mechanical injury was best seen. By pressure of the fingers on the web, partial stagnation was produced in many of the vessels; and when this yielded to the returning current, the walls of the vessels were seen studded with the white globules; whilst many others of the same kind rolled over them slowly in the direction of the current. A similar result ensued after the web had been stimulated by capsicum or an aromatic water. Even in the rapid flow of blood following these applications, minute globules could be seen creeping slowly along the transparent outline of the larger vessels; and as the arteries contracted, and the flow through the other vessels became less rapid, the number of these globules increased, their motion became slower, and many adhered to the sides of the vessels. If the stimulus used was rather strong or long applied, the number of sticking globules was so great as to prevent the red particles from passing; and these becoming impacted in increased numbers, gave to the obstructed vessels a uniform and deeper red colour. When the stimulation was moderate, and equally applied to the web, the stagnation usually took place first in some of those anastomosing veins, in which the current is naturally slow, and varying in direction; but when a stronger stimulus (as an essential oil) was used, the stagnation speedily ensued at the point of its application; in fact, unless very minute quantities were employed, the stagnation was almost immediate and extensive.

"I have varied these observations in a great many ways, and I have always found considerable or continued irritation of the vessels (textures) of the frog's web to be attended with the appearance and adhesion of the colourless globules; and that, when the irritant used is at all strong, or frequently applied, many vessels become totally obstructed, appear larger and redder than before, by the accumulation of red particles in them, (the blood-liquors having passed on,) and exhibit to the naked eye all the appearance of inflammatory injection. The chief cause of obstruction seems to be comprised in the two circumstances, the increased production of the white globules, and their remarkable disposition to adhere to the walls of the vessels, and to one another." (pp. 209-11 )

Dr. Williams is of opinion that the white corpuscles are *newly formed*, immediately upon the application of an irritant; and that, from the suddenness of their appearance, they cannot be regarded as *cells*. The researches of Mr. Addison, however, have clearly proved (to our minds at least) that they are true cells; and when it is considered how very small a proportion of the whole circulating system can be placed under observation at once, it does not appear to us difficult to account for their rapid accumulation in an irritated part, without imagining that they are suddenly generated. For there is no evidence that their quantity is increased in the whole mass of the blood, until the irritation has subsisted for some time, during which they are rapidly undergoing the process of cell-multiplication,—whilst at the same time, (according to the view we have adopted,) and by the very same act, increasing the quantity of fibrin in the blood.

Following out his opposition to the doctrine of "vital attraction" in all its forms, Dr. Williams objects to the idea that the adhesion of the white corpuscles to the walls of the vessels is anything else than a physical phenomenon.

"The reason why the red particles are more readily carried in the stream, appears to be, that they expose a large surface to the current; and being covered by a perfectly smooth unadhesive membrane, they are not liable to stick to the walls. The white globules, on the other hand, are more compact; and although, when in the current, are readily carried by it, when more out of it, and in the motionless layer, are merely rolled by it, like pebbles in a rapid stream. Further, they manifest a distinctly adhesive property, which causes them to stick to the walls of the vessels. In this respect they contrast remarkably with the red blood-discs; and the newly-formed globules of irritated vessels seem to have this adhesive property in the highest degree; they are probably without a covering." (p. 212.)

Now we must take leave to remark that this idea of the possession, by the white corpuscles, of a "distinctly adhesive property," by which we presume that Dr. Williams means, in common parlance, that their surface is sticky, is a mere assumption, and is unsupported by any evidence whatever. For when inflamed blood is drawn from the vessels, the numerous white corpuscles which it contains do not show any tendency to adhere to each other, or to the red particles, which they would naturally do, if their surfaces were adhesive enough to cause them to stick to the walls of the vessels. On the contrary, they are distinguished by their isolation; whilst the *red* corpuscles *do* show a tendency to adhere to one another in rolls, especially in inflammatory blood; in spite of the smoothness of surface, which is dwelt on by Dr. Williams, as explaining their non-adhesion to the walls of the vessels. Now it is generally conceded that this attraction is a consequence of their peculiar vital endowments; and until the existence of some other cause shall be proved, we think that there is a strong argument from analogy in favour of the *vital* nature of the attraction between the white corpuscles and the walls of the blood-vessels. The last clause of the paragraph just quoted is not supported by any observations; and is opposed to all that we *know* of the nature of the white corpuscles.

As to the phenomenon itself, however, there is no difference of opinion between ourselves and Dr. Williams; and we avail ourselves of his figure and description to explain to our readers the condition of the blood and of the vessels in an inflamed part.

"The accompanying diagram exhibits the appearance of a small portion of the capillaries of a frog's web, after the application of a grain of capsicum. The elliptical blood-discs (*b*) are running in the axis of the vessel, which is much narrowed by white globules adhering to the walls, or only slowly rolling along them. These globules are speckled with nuclei or granules, refract the light strongly, and when rolled on by the current, some of them become pear-shaped from their sticking to the vessel, thus forming a kind of dragging tail, seen very well on those marked (*a*); on altering the focus, globules may be seen adhering to the other parts of the vessel. The shaded portion (*c*) is totally obstructed with lymph- and blood-particles, so impacted together, as to form a homogeneous red mass. In such a case I have often seen the particles at (*d*,) exhibit a pulsating or oscillatory motion, (corresponding with the action of the heart;) and this, after a time, succeeds in breaking down the obstructing mass, which passes away in clots, leaving the vessel (*c*) studded with lymph-globules like the other." (p. 243.) To the accuracy of this figure and description, we have the additional testimony of Mr. Addison. With the following induction, we fully accord:

"It seems, then, to be well established, that an essential part of inflammation is the production of numerous white globules in the inflamed vessels; and that the obstruction of these vessels is mainly due to the adhesive quality of these globules. The production of these globules must probably be considered as an ultimate fact in the history of inflammation and nutrition; but it may be observed, that sometimes it seems to be the direct effect of an irritant acting on the blood-vessels and their contents; in other instances, it seems rather to result from determination of blood into previously congested capillaries. Any circumstance causing continued determination of blood where congestion is already present, will occasion the production of the white globules, and consequently inflammatory obstruction may ensue. The complete obstruction of some capillaries by coagulation takes place in all cases of severe inflammation of the frog's web; but there are slighter kinds of increased vascularity, in which there is no total obstruction, but a continued enlargement of the capillaries and veins, as well as of the arteries. This might be called simple determination of blood; but it differs from that of a transient character, in the motion of the capillaries and veins being slower, and in the vast number of white globules seen moving slowly in them. Very probably this kind of process takes place in the lowest forms of inflammation, and increased nutrition independent of inflammation. Something of this kind is generally seen in the capillary circulation of young frogs." (pp. 213-4.)

If Dr. Williams had connected with the development of a large amount of additional white corpuscles, the production of an increased quantity of fibrin, we believe that he would have come very near the truth, in regard to the participation of the blood in the phenomena of inflammation. We agree with him in thinking that both these phenomena may be present, to a moderate degree, without the existence of inflammation; since, whenever the formative processes are peculiarly active, as in young animals, and in the generation of new tissue for the reparation of injuries, they can only be supplied by such a modification in the character of the blood.

As to the *depression of the proper formative powers* of the solid tissues supplied by the blood, in the state of inflammation, we cannot conceive that much difference of opinion can exist, when the phenomena adverted to in our summary are duly considered; and we shall therefore proceed to the next head, the *altered motion* of the blood in the vessels of an inflamed part; in regard to which phenomenon we believe that there is little or no difference of opinion, although various causes have been assigned to it. Some, indeed, have regarded it as the essential character of inflammation; and even Dr. Williams falls into this error, for such we must term it. We cannot consider as the essential character of a morbid state that which is only *one of the results* of antecedent changes; on the united effects of which the various phenomena of that state depend, as we think may be shown in regard to the altered motion of the blood in inflammation. It is the tolerably uniform result of observation, that the application of a moderately stimulating substance to the web of the frog, *first* produces an accelerated motion of the blood in a part, with contraction of its vessels. Now we take these two results to be due to the temporary *exaltation* of its vitality; which will produce contraction of the vessels, by the excitation of their tonicity; and more rapid motion of the blood through them, by the more energetic demand for it. If, as Dr. Williams seems to suppose, diminution of the caliber of the arteries induces a more rapid rate of motion through them as a *physical* result, why does not this happen when cold is applied to a part? We know that it is quite otherwise, the arteries being contracted, in virtue of their excited tonicity, to which cold is a specific stimulus;* whilst the rate of

* It may be objected to this view, that if the vitality of the tissues in general be lowered by cold, and increased by heat, that of the contractile coat of the arteries ought to folmotion through them is slow, because the general vitality of the surrounding tissues is lowered. On the other hand, moderate warmth induces enlargement of the arteries with accelerated motion; because, whilst it relaxes the tonic contraction, it renders the formative processes more energetic, and thus increases the demand for blood.

But when the stimulating application has been applied for some time, or is more powerful in the first instance, an opposite result succeeds. The arteries dilate, and the blood moves more slowly; and this retardation may reach the point of complete stagnation. At the same time, there is increased motion of the blood in the vessels of the *surrounding* tissue, which, as well as the trunks leading to the inflamed part, are dilated; so that the whole quantity proceeding through the organ is increased. Now this dilatation is probably due in great part to the depressed vitality, or defective tonicity of the arteries, as Dr. Williams suggests; but we cannot (with Dr. Macartney and others) suppose this to be an essential character of inflammation; since it appears to us merely a consequence of the diminished vital power of the whole texture, which we believe to be one of its chief elements. The retardation of the blood in the central portion seems to us to manifest itself at an earlier period than could be accounted for on the principle of mechanical obstruction, caused by adhesion of the white corpuscles to the walls of the vessels, in the manner described by Dr. Williams; and we should explain it on the principle we have so frequently alluded to, that of diminished capillary power, (by which we mean, not mechanical force, but the force generated by the reactions taking place between the blood and the tissues,) resulting from the depressed state of the formative processes.* When the adhesion of the white corpuscles takes place to any extent, however, it undoubtedly becomes an additional cause of obstruction; and has probably a large share in producing the complete stagnation which is frequently to be observed. And this stagnation, when once produced, will of course act unfavorably upon the surrounding solids; occasioning a still further depression of their vitality.

We have lastly to consider the *results* of the inflammatory process; and these may, we think, be extremely well accounted for, upon the views we have been upholding. The mere accumulation of blood, and its stagnation in the vessels of the inflamed part, will give rise, as in the case of congestion, to the transudation of the *serous* portion of the circulating fluid; a physical action, to which an increased disposition is probably low the same rule; and that cold ought to produce dilatation, and heat to occasion contraction of their caliber. But it is to be recollected, that *cold* acts as a *specific stimulus* to the *tonicity* of the fibrous tissue, in the same manner as nervous influence or galvanism calls into action their contractility.

* By Dr. Alison it is believed that the retardation in the flow of blood is due to an *increased attraction* between its elements and the tissues. Such an increase would certainly appear to exist, especially in regard to the white corpuscles; but we doubt whether the supposition is necessary. In the normal process of nutrition, each particle seems to be attracted towards a certain point, where it is to undergo a change, and to be repelled from it after having undergone that change;—just as bodies are alternately attracted and repelled by another which is charged with electricity. Now on our view of the nature of Inflammation, the *attraction* of the particles of blood to the tissues still exists; but it does *not* give place to *repulsion*, in consequence of the absence of those changes to which it ought to be subservient; and therefore adhesion of its particles to the walls of the vessels manifests itself.

given, by the weakened state of the walls of the vessels. When the proportion of fibrin begins to increase, and the formative power of the part, together with the firmness of the walls of the vessels, diminish still further, there is a tendency to the effusion of the *fibrinous* portion of the blood also. As the experiments of Mr. Robinson* have proved that this may be poured forth, without the effusion of the red particles, under the influence of physical causes only, we do not think it necessary to invoke the aid of any other, under the various favouring conditions which we have specified. When the fibrin is thus effused, it will occasion a consolidation of the tissue, into the interstices of which it is poured; if it be sufficiently plastic to undergo spontaneous conversion into an organized structure. If poured out upon a membranous surface, it will form a false membrane; the structure of which is exactly the same as that of the buffy coat of the blood, only more perfect. The "exudation-cells" found in such tissues may be supposed to originate in the minute granules or cell-germs, which are seen floating in the liquor sanguinis of the circulating blood, and which may be considered as the offspring of the white corpuscles; but this is merely a probable hypothesis, chiefly supported by the fact that, as far as we *know*, all cells are developed from germs supplied by preexisting cells. If largely diluted with serum, the fibrin may be effused in a liquid form into a cavity, (as that of a serous sac,) and occasion the spontaneous separation of its contents into clot and serum. But if the fibrin be not duly elaborated, which may happen in consequence of a peculiar state of the system induced by various causes, it will be effused in various *cacoplastic* or *aplastic* forms, which we need not here stop to describe, particularly as they are well detailed in Dr. Williams's treatise.

The effusion of fibrinous matter, however, in the aplastic form of *pus*, must be ranged under a different category; being due to a *local* action more frequently than to a constitutional disorder; and into the causes of this we must next inquire. We cannot ascribe the suppuration of an ordinary *phlegmon*, to anything peculiar in the state of the blood; because we find no change in its condition, even when this process is actively taking place, the fibrin being still present in large amount, and of a high degree of plasticity. Moreover, at the very time at which the effusion of pus is taking place in one spot, we have (in subjects of a healthy character, at least,) an effusion of organizable fibrin into the surrounding tissue, producing its consolidation, and limiting the purulent effusion. Hence it appears to us next to certain, that the pus must be formed in the spot where it is effused; excepting in cases where there are diffused deposits of it, not preceded by inflammation in the parts so affected, which class of cases we shall presently consider. If our doctrines be correct, the formation of pus is due to an extremely depressed state of the vitality of the part most inflamed; which will exert an unfavorable influence upon the vital properties of the effused liquor sanguinis. Of such an operation there is no want of examples; the changes produced in the circulating blood by miasmata or infectious matters, in very small quantity, are "pregnant instances." The researches of Mr. Addison have rendered it probable, that the *liquor puris* is a degraded or degenerated form of the

* Medico-Chirurgical Transactions.

liquor sanguinis; and though we cannot agree with him in considering the pus-corpuscles as metamorphosed white corpuscles, (because we cannot think it probable that bodies so large as these can pass out of the capillaries, without an absolute rupture of their walls,) we are inclined to think that the two are developed from the same germs, the minute molecules circulating in the blood, and continually regenerated, (like the germs of the simple cellular plants,) by the bursting of the parent corpuscles. These germs, developed in the midst of a degenerated fluid, will have a degraded form; and thus from healthy liquor sanguinis, and from cell-germs, which would have otherwise produced exudation-corpuscles, will be produced, under the deteriorating influence of the contact of a dying and disintegrating tissue, liquor puris and pus-corpuscles. This view is borne out by the well-known fact that the admission of air to a surface, on which the formation of new tissue is taking place, will cause suppuration to occur, where no pus was previously effused; for the contact of cold air, depressing the vitality of the superficial tissue, causes a corresponding deterioration of the effused product; which thus, instead of becoming organized into new tissue, is converted into aplastic pus.

The circumscription of the deposit of pus in a solid tissue, by a fibrinous effusion, is well explained on the foregoing theory. For, as the inflammatory process is most intense in the centre, and gradually diminishes towards the periphery of the affected part, it will be in the *centre*, where the stagnation is most complete, and the vitality of the tissues the most depressed, that the production of pus will take place; whilst in the surrounding parts, the fibrinous effusion, not being subjected to this unfavorable influence in the same degree, will become more or less organized, and will prevent the extension of the purulent deposit. But when unfavorable states of the constitution, produced by general depressing causes of various characters, or by poisons directly introduced into the blood, prevent the formation of *perfect* fibrin, we may then have effusion of pus in an inflamed part without any such restriction; and when once generated, it will act unfavorably upon the surrounding parts, whose vitality is already depressed, causing their disintegration, and a consequent spread of the purulent effusion. This seems to us to be the true theory of *asthenic* inflammation; and the rationale of the success of a *general* tonic, or even stimulating plan of treatment, even whilst *local* depletion or sedatives are employed. Moreover, where pus is absorbed into the circulation,* there is good reason to believe, even from observation of its effects upon recently drawn blood, that it will occasion a most deleterious change in the character of the fluid; at once impairing its coagulability, altering the condition of its corpuscles, and giving rise (like a ferment) to an increased tendency to the production of purulent matters. When this is circulating with the blood, it may be separated by the two great depurating organs, the lungs and liver; or may be deposited in other parts, without the occurrence of inflammation in them; an increased tendency to such deposits in a part being perhaps induced by local congestion, which gives a predisposition to an effusion of some kind.

We quite agree with Dr. Williams in thinking that *ulceration* is only

* There are cases, it is true, of the apparent absorption of pus, in which these results do not manifest themselves; but it is probable that in these it undergoes a change, before entering the circulating current.

a modification of the ordinary suppurative process. It is generally accompanied with some degree of purulent effusion; but this commences rather on the surface, than in the substance, of an organ. The great feature in both is the depression of vitality, and gradual disintegration of tissue, depending upon the retardation or stagnation of the current of blood through it; and the chief difference seems to be, that there is not the same increase of plasticity in the blood, and tendency to effusion from it, in the early stage of the ulcerative process, as in the suppurative. It is not until a thick creamy pus is exuded from an ulcer, manifesting, not only an improved condition of the part itself, but an increase in the plasticity of the liquor sanguinis, of which it is a modified form,—that the healing process is fairly established; and, in order to accomplish this change, such constitutional means are required as shall favour the increase in quantity, or render the elaboration more perfect, of the fibrin of the blood; whilst local stimulating applications may have the same effect, by exciting the true inflammatory process, which was previously deficient.

When *gangrene* results from acute inflammation, it may be easily traced to the complete stagnation of blood in the vessels of a part; and the consequent loss of that vitality which was previously depressed. And the same process, taking place to a more moderate degree in the surrounding parts, will cause that deposition of organizable lymph, which limits the extension of the destructive process, and covers the surface from which the slough separates; and when, as in the case of diffused suppurative inflammation, there is not plasticity enough in the blood for this circumscribing effusion to be organized, the gangrene spreads, by the ordinary cause, i. e., the decomposing influence, upon a living part whose vitality is already depressed, of destructive changes going on in its immediate neighbourhood. And this want of limitation we see especially in cases where the gangrene does not result from local inflammation, but from some general depressing cause, which lowers the power of the whole system, whilst acting upon some one part especially. It is here necessary, as in diffusive suppuration, to take measures to *increase* the plasticity of the blood, instead of diminishing it; in fact, to excite a *sthenic* inflammatory condition, which did not exist previously. For if the views we are propounding be correct, the difference between the *sthenic* and *asthenic* forms of inflammation consists essentially in this, that, whilst in both there is a depressed vitality of the solid tissues affected, there is in the former a great increase in the plasticity of the blood, causing a tendency to effusion of organizable lymph, or of its modifications; which tendency is deficient or imperfect in the latter, in consequence of a want of the due elaboration of the fibrinous element of the blood. The distinction is most important in practice; because it leads us to see that, as the production of fibrin is necessary for reparation as well as for the original formation of tissue, we must carefully watch for the indications of its presence in sufficient or insufficient amount; and regulate our general treatment accordingly.

Our object in the preceding pages has been to show, that, starting from the two fundamental positions which we have assigned as the characteristic or essential conditions of inflammation, (at least in its sthenic form,) namely, a diminished vitality or formative power in the solid tissues on the one hand, and an increased plasticity of the blood on the other; we may account for all the leading phenomena of this process, upon simple physiological principles. The impaired tonicity of the *vessels* permits their enlargement. The depression of the ordinary vital actions of the part occasions *the diminution in the rate of the blood's movement;* which effect is aided by the adhesion of the white corpuscles to the sides of the vessels, producing mechanical obstruction. The stagnation, partial or complete, of the blood, produces in its turn *an increased depression* of the vitality of the tissues, and a tendency to the *effusion* of its fluid parts. The increase in the proportion of fibrin in the blood, together with the weakened state of the walls of the vessels, give to the *inflammatory effusion* the characters of the *liquor sanguinis,* rather than those of the *serum.* If the tissues into which this is poured sufficiently retain their vitality, the effusion becomes partially organized, filling up their interstices, and producing *consolidation.* Or if it be effused upon a surface, whose vital properties have not been too much lowered, it will form a *new membrane* of considerable tenacity. But if poured out into a tissue, or upon a surface, whose vitality is very much depressed, it is so far changed as to present the unorganizable form of *pus.* At the same time the tissue itself undergoes disintegration, in consequence of its impaired vitality; and its parts are removed by interstitial absorption (if this process still continues); or they are dissolved in the purulent effusion; or the whole mass is thrown off together in a slough.

In the *asthenic* form of inflammation, on the other hand, the same loss of vitality, suppuration, ulceration, or gangrene may occur; but there is little or no tendency to the restriction of these processes to one spot, or to the reparation of the injuries which they effect; the fibrinous material being either deficient in quantity or in plasticity. Hence we see that, in sthenic inflammation, as in the reparative processes, the increased production of fibrin in the blood is to be regarded as really a conservative operation. If it may be attributed (as we have endeavoured to prove) to the increased generation of white corpuscles, it seems to us that we make an important step in the analysis of this complex assemblage of phenomena. But the question still remains, What occasions this increased generation? To this we shall not at present attempt to make any reply; since we are in want of data, on which to found even a probable speculation. But we think it is something to be able to point to a fact so definite as this, as one of the essential components—if not *the* essential one—of the process under discussion.

We have not touched upon certain chemical views which have lately been propounded, in regard to the changes which the blood undergoes in Inflammation, by the increased *oxidation* of the protein compounds;—not because we do not regard them as possessing a high degree of interest, but because we deem them as yet too crude and unsettled to be combined, in any view of the subject, with facts which have been more satisfactorily ascertained; and because they belong to an entirely distinct category, and are quite subordinate to those we have been discussing. Supposing it to be true that this increase takes place, it is only another point of difference between healthy and inflammatory blood—the *essential* difference of which, in reference to the vital processes of nutrition and inflammation, consists in the increased plasticity of the latter.

## Figures and Tables

**Figure f1:**